# Another host, another mystery: Infection of polychaete *Pista palmata* (Annelida: Terebellidae) with a new blood fluke (Digenea: Aporocotylidae) in the coastal Northwest Atlantic Ocean

**DOI:** 10.1051/parasite/2026048

**Published:** 2026-09-08

**Authors:** Anna N. Chowansky, Kristina M. Hill-Spanik, Dennis E. Kyle, Isaure de Buron

**Affiliations:** 1 Department of Biology, College of Charleston 205 Fort Johnson Road Charleston SC 29412 USA; 2 Center for Tropical & Emerging Global Diseases, University of Georgia Athens GA USA

**Keywords:** Intermediate Host, Western Atlantic Ocean, Microcercous cercaria, Schistosomatoidea, Aporocotylidae

## Abstract

Terebelliform annelids can serve as intermediate hosts for blood flukes of marine fishes and sea turtles. The scarcity of global reports on the life cycles of these parasites reflects the need for further exploration. Our objective was to further our knowledge of the diversity and life cycles of blood flukes in coastal South Carolina (SC), United States. Four species of terebellids (*Enoplobranchus sanguineus*, *Amphitrite ornata*, *Pista palmata*, and *Streblosoma hartmanae*) were collected (total *N* = 201) at low tide from September 2025 to February 2026 at two localities in SC. Twelve specimens of three terebellid species were infected, and parasites were identified by sequencing the LSU and ITS2 regions of ribosomal RNA and the COI mitochondrial DNA gene. *Enoplobranchus sanguineus* (12.9% prevalence, 4/31) and *A. ornata* (3.6% prevalence, 2/55) were infected with the previously reported blood flukes *Neospirorchis* sp. Neogen14 (Unicaecidae) and *Cardicola parvus* (Aporocotylidae), respectively. No specimens of *S. hartmanae* were infected (0/86). One new aporocotylid with mature sporocysts containing microcercous cercariae was discovered in *P. palmata* (20.7% prevalence, 6/29). Phylogenetic analysis revealed that sequences of the new aporocotylid were in a well-supported clade with sequences of *Braya* spp. and *Cardicola abu*. The fish host of this parasite has yet to be discovered to fully elucidate the life cycle of this new aporocotylid.

## Abstract (fr)

Les annélides térébelliformes peuvent servir d'hôtes intermédiaires pour des trématodes sanguins parasitant des poissons marins et des tortues marines. La rareté des données mondiales concernant les cycles biologiques de ces parasites justifie de poursuivre les recherches. Notre objectif était d'approfondir les connaissances sur la diversité et les cycles biologiques des trématodes sanguins dans les zones côtières de la Caroline du Sud (CS), aux États-Unis. Quatre espèces de Terebellidae (*Enoplobranchus sanguineus*, *Amphitrite ornata*, *Pista palmata* et *Streblosoma hartmanae*) ont été récoltées (effectif total *N* = 201) à marée basse, entre septembre 2025 et février 2026, sur deux sites de CS. Douze spécimens appartenant à trois espèces de térébellidés étaient infectés, et les parasites ont été identifiés par séquençage des gènes d'ARNr (régions LSU et ITS2) et du gène mitochondrial COI. *Enoplobranchus sanguineus* (prévalence 12,9 % ; 4/31) et *A. ornata* (prévalence 3,6 % ; 2/55) étaient infectés respectivement par *Neospirorchis* sp. Neogen14 (Unicaecidae) et *Cardicola parvus* (Aporocotylidae), deux espèces déjà connues. Aucun spécimen de *S. hartmanae* n'était infecté (0/86). Un nouvel Aporocotylidae, présentant des sporocystes matures contenant des cercaires de type microcerque, a été découvert chez *P. palmata* (prévalence 20,7 % ; 6/29). L'analyse phylogénétique a révélé que les séquences de ce nouvel Aporocotylidae s'inscrivaient dans un clade bien soutenu aux côtés des séquences de *Braya* spp. et de *Cardicola abu*. L'hôte poisson de ce parasite reste à découvrir pour élucider entièrement le cycle biologique de ce nouvel Aporocotylidae.

## Introduction

Blood flukes in the family Aporocotylidae Odhner, 1912 are known for being pathogenic to marine finned fishes, in which they can induce endocarditis or meningitis [1, 33, 41, 59]. Aporocotylidae is a diverse and speciose family with a worldwide distribution [57], and eight species, six of which are *Cardicola* Short, 1953 [46], are known from their fish definitive hosts in the Northwest Atlantic Ocean and in the Gulf of Mexico. In Canada, *Aporocotyle simplex* Odhner, 1900 was reported from three fish hosts: the American plaice *Hippoglossoides platessoides* (Fabricius) (see [44]), the roundnose grenadier *Coryphaenoides rupestris* Gunnerus, and the roughhead grenadier *Macrourus berglax* Lacepède (see [64]), although the blood flukes in the grenadiers (Gadiformes) may have been misidentified, as *A. simplex* is thought to be specific to Pleuronectiformes [25, 26, 53]. On the Atlantic coast in the United States (USA), all known aporocotylids have been reported from southeastern coastal fishes from North Carolina to Louisiana: *C. laruei* Short, 1953 [46] was reported from the sand seatrout *Cynoscion arenarius* Ginsburg (see [46]) and the spotted seatrout *Cy. nebulosus* (Cuvier) (see [3, 34, 46]), *C. forsteri* Cribb, Daintith, & Munday, 2000 [12] from the Atlantic bluefin tuna *Thunnus thynnus* (L.) (see [7], reported as northern bluefin tuna), *C. palmeri* Bullard & Overstreet, 2004 [8] from the black drum *Pogonias cromis* (L.), *C. currani* Bullard & Overstreet, 2004 [8] from the red drum *Sciaenops ocellatus* (L.) (see [8]), *C. parvus* Bullard, Baker & de Buron, 2012 [6] from Atlantic croaker *Micropogonias undulatus* (L.) and spotted seatrout *Cy. nebulosus* (see [6]), *C. langeli* Bullard, 2013 [5] from sheepshead *Archosargus probatocephalus* (Walbaum) (see [5]), and *Mugilitrema labowskiae* Warren & Bullard, 2024 (in [58]) from white mullet *Mugil curema* Valenciennes (see [58]). Aporocotylids are known to use annelid polychaetes as intermediate hosts, particularly terebellids [9, 25, 26, 39, 45, 50, 51]; however, for the eight species mentioned above, only three life cycles are known, with two being from coastal South Carolina (SC), USA [47]: *C. laruei* uses *Terebella lapidaria* (L.), and *C. parvus* uses *Amphitrite ornata* (Leidy) and *Enoplobranchus sanguineus* (Verrill) (see [47]). The life cycle of *A. simplex* was elucidated in the North Sea [25] and possibly in Iceland [26]*.* Given the apparent abundance of blood flukes in southeastern US coastal fishes, our goal was to discover other intermediate hosts in this area, and in SC in particular. Herein, we report the finding of yet another novel aporocotylid and cercaria type for our coast in a terebellid polychaete, *Pista palmata* (Verrill).

## Materials and methods

### Collection of annelids and parasites

Annelids were collected from September 2025 to March 2026 by hand from two localities in SC, USA: North Inlet (33.327°N, 79.208°W) and Charleston Harbor (32.754°N, 79.895°W). Annelids were kept alive in aerated seawater, sorted per morphotype, and examined within four days of collection. Specimens with obvious infection were isolated into individual dishes to collect parasites. Individual annelids were identified morphologically [27]. Because we were familiar with *E. sanguineus* and *A. ornata* (see [16, 47]) but not with the two other terebellid species encountered in this study, infected individuals of *P. palmata* were fixed in 100% ethanol (EtOH) for later molecular confirmation of host identification. Sporocysts and cercariae were isolated, washed in filtered sea water, and either photographed live or fixed in 5% formalin or 100% EtOH, with some being heat killed prior to fixation.

### Morphological study

Parasite specimens were processed for either scanning electron microscopy (SEM) or light microscopy (LM). For SEM, specimens were mounted on poly-L-lysine coated coverslips, dehydrated in a graded series of EtOH, chemically dried using hexamethyldisilazane, sputter coated with gold-palladium, and examined with a JEOL JSM-IT510 electron microscope operating at 5 to 10 kV. For LM, specimens were stained with acetocarmine, dehydrated in a series of EtOH concentrations, cleared in methyl salicylate, and then mounted in Canada balsam prior to being examined and measured by using an ocular micrometer on a compound microscope. Voucher parasite and host specimens were deposited at the Harold W. Manter Laboratory University of Nebraska, Lincoln, NE, USA under the numbers HWML 218489-218493 and HWML 119334, respectively, and at the Muséum National d’Histoire Naturelle, Paris, France under the numbers MNHN-HE-139YT-144YT.

### Molecular study

We used DNA sequencing to identify the parasites encountered from each of the 12 individual annelids found to be infected, as well as the six infected annelids previously unknown to us and morphologically identified as *P. palmata*. DNA of blood flukes and hosts was extracted using a DNeasy Blood and Tissue kit (QIAGEN, Hilden, Germany) based on the manufacturer’s protocol after initial digestion with proteinase K at 37 °C overnight. For blood fluke identification, we targeted a portion of the large subunit ribosomal RNA (LSU rRNA) gene. If the LSU rRNA gene sequence did not provide a species-level identification, we then amplified and sequenced the second internal transcribed spacer region (ITS2) of the rRNA array and a portion of the mitochondrial cytochrome *c* oxidase I (COI) gene for PCR and sequencing. For the annelids, a portion of the COI gene was targeted. Primers and their sequences are provided in Table 1. Sequences generated from this study were deposited in GenBank (Table 2).

**Table 1:** Primers used in PCR and sequencing of a portion of the large subunit ribosomal RNA (LSU rRNA) gene and the second internal transcribed spacer region (ITS2) of the rRNA array of blood flukes, and the mitochondrial cytochrome *c* oxidase I (COI) gene of both blood flukes (Parasite) and terebellids (Host). Primers JB3 + COI-R were used to amplify and sequence the COI gene of *Neospirorchis* sp., and Dig_cox1Fa+Dig_cox1R were used to amplify the COI of the new blood fluke found herein.

Target	Gene region	Primer name	Direction	Primer sequence (5′–3′)	Product Size (~ bp)	Reference
Parasite	LSU	digl2	F	AAGCATATCACTAAGCGG	1,400	[54]
		1500R	R	GCTATCCTGAGGGAAACTTCG		[55]
		ECD2*	R	CTTGGTCCGTGTTTCAAGACGGG		[30]
	ITS2	GA1	F	AGAACATCGACATCTTGAAC	390	[2]
		ITS2-2	R	CCTGGTTAGTTTCTTTTCCTCCGC		[10]
	COI	JB3	F	TTTTTTGGGCATCCTGAGGTTTAT	800	[4]
		CO1-R	R	CAACAAAATCATGATGCAAAAGG		[35]
	COI	Dig_cox1Fa	F	ATGATWTTYTTYTTYYTDATGCC	475	[60]
		Dig_cox1R	R	TCNGGRTGHCCRAARAAYCAAAA		[60]
Host	COI	jgLC01490	F	TNTCNACNAAYCAYAARGAYATTGG	690	[18]
		jgHCO2198	R	TANACYTCNGGRTGNCCRAARAAYCA		[18]

^*^Internal primer that was only used for sequencing.

**Table 2:** GenBank accession numbers of large subunit (LSU) ribosomal RNA (rRNA) gene, second internal transcribed spacer (ITS2) region rRNA, and mitochondrial cytochrome *c* oxidase I (COI) DNA sequences resulting from this study.

Species	Isolate number	LSU	ITS2	COI
Aporocotylidae sp. 1 *ex Pista palmata* SC	x150_012426	PZ725571	PZ735424	PZ761205
	x160_012426	PZ725572		PZ761206
	x03_020626		PZ735425	
	x04_020626		PZ735426	
	x05_020626		PZ735427	
*Cardicola parvus*	x03_110525	PZ745526		
	x10_110525	PZ745525		
	x12_110525	PZ745524		
*Neospirorchis* sp. Neogen14	x07_110525	PZ745500		
	x01_012026	PZ725573		PZ754648
	x02_012026	PZ725574	PZ748315	PZ754649
	x03_012026	PZ725575	PZ748316	PZ754650
*Pista palmata*	x170_012426			PZ760218
	x180_012426			PZ760219
	x06_020626			PZ760220
	x07_020626			PZ760225
	x08_020626			PZ760226
	x09_020626			PZ760221

For the parasite LSU and COI PCR assays, a 25-μL total reaction contained 1× Promega GoTaq Flexi PCR Buffer (Madison, WI, USA), 1.5 mM MgCl_2_, 0.25× Rediload (Invitrogen, Waltham, MA, USA), 0.2 mM dNTPs, each primer at 0.5 μM, 1 U Promega GoTaq^®^ DNA polymerase, and 2 μL template DNA. For the LSU PCR, cycling conditions were as follows: 94 °C for 3 min, followed by 40 cycles at 94 °C for 30 s, 55 °C for 30 s, 72 °C for 1 min, and final extension at 72 °C for 7 min. Cycling for the parasite COI PCR was similar except that the initial denaturation was only for 1 min, only 35 cycles were done, and the annealing temperature was 45 °C. For amplification of the parasite ITS2 region of the rRNA gene, the final concentrations of the reagents were the same as above, but 3 μL of template DNA was used, and cycling was as follows: 15 s at 94 °C followed by 32 cycles at 94 °C for 30 s, 56 °C for 30 s, 68 °C for 51 s, and then by a final extension at 68 °C for 3 min. To amplify the COI of terebellid hosts, the reagent final concentrations were the same as the LSU and COI PCR assays above, except that MgCl_2_ was 2 mM and primers were 0.3 μM, and 3 μL template DNA was used. Cycling was as follows: 95 °C for 5 min, 35 cycles of 95 °C for 30 s, 48 °C for 30 s, 72 °C for 45 s, and final extension at 72 °C for 5 min.

PCR products, including positive and negative controls, were electrophoresed on 1% agarose gels stained with GelRed (Biotium Inc., Hayward, CA, USA) and visualized under UV light. Products were purified using ExoCleanUp FAST (VWR Life Science, Radnor, PA, USA) and sent to Eurofins MWG Operon LLC (Louisville, KY, USA) for direct, bi-directional sequencing using the same primers used for PCR, except for the LSU rRNA gene, for which an internal sequencing primer was also used (Table 1).

Sequencher v 5.4 (Gene Codes Corporation, Ann Arbor, MI, USA) was used to assemble and edit contiguous sequences, which were then compared to those in the NCBI GenBank database using megaBLAST [36]. If none of the three markers yielded a species-level identification (i.e., BLAST results were < 99% similar), we generated alignments with the GenBank sequences with which our sequences were most similar based on our BLAST results (Table 3). The LSU rRNA and COI gene sequence alignments were done using ClustalW in MEGA12 [28], and the ITS2 sequence alignment was done using MAFFT v. 7 [23] accessed at NGPhylogeny.fr [29]. The ITS2 alignment was further curated using BMGE (Block Mapping and Gathering with Entropy [13]) at NGPhylogeny.fr [29]. *P*-distance calculations were done in MEGA12 [28] using the partial deletion option. We also included sequences of *C. orientalis* in the alignments used to calculate *p*-distance, as cercariae of this species most closely resembled those we found in *P. palmata* (see [45]) (Table 3).

**Table 3:** GenBank accession numbers of large subunit ribosomal RNA (LSU rRNA), second internal transcribed spacer region (ITS2) rRNA, and mitochondrial cytochrome *c* oxidase I (COI) gene sequences from aporocotylid species used in calculating *p*-distances and phylogenetic analysis.

Species	LSU	ITS2	COI
*Braya jexi*	MZ264863	DQ059624	OM234758
*Braya psittacus*	MZ264864	DQ059625	MZ295260
*Braya yantschi*	MZ264865	DQ059628	OM234733
*Cardicola abu*	MH161379	MH161380	MZ295263
*Cardicola bullardi*	KX523190	KX523191	MZ295264
*Cardicola forsteri*	KT119353	AB742428	MZ295265
*Cardicola orientalis* ^*^	KT119355	AB742427	PV670779
*Cardicola nolani*	OM236519	OM236529	OM234740
*Rhaphidotrema kiatkiongi* ^†^	MZ264868	MZ264861	MZ295270
*Spirocaecum covacinae* ^†^	MF140283	DQ059634	MZ295272

^*^Included only in alignments used to calculate *p*-distances. ^†^Used as outgroup sequences for phylogenetic analysis.

Alignments of all three markers (LSU, ITS2, and COI) were concatenated in MEGA12 [28] for subsequent phylogenetic analysis. Maximum parsimony analysis using 1,000 bootstrap replicates and the Subtree-Pruning-Regrafting (SPR) algorithm [35, 37] with 100 random additions was done in MEGA12 [28]. Bayesian analysis was performed using MrBayes v. 3.2.7_0 [20] accessed at NGPhylogeny.fr [29]. (GTR+I+G; 2 runs, 4 Markov chains, 10 million MCMC generations, sample frequency = 1,000). Ten thousand trees were produced, with 25% removed as burn-in. Sequences of *Spirocaecum covacinae* and *Rhaphidotrema kiatkiongi* were used as the outgroup based on Cutmore and Cribb [14].

## Results

### Prevalence of infection and general observations

In total, 201 specimens of four species of terebellid polychaetes were collected and examined. Out of the four species, *A. ornata* (*N* = 55), *E. sanguineus* (*N* = 31), *P. palmata* (*N* = 29), and *Streblosoma hartmanae* Kritzler (*N* = 86), a total of 12 annelids were infected with sporocysts of blood flukes, which were identified molecularly (see below): four individuals of *E. sanguineus* were infected with *Neospirorchis* sp. Neogen14 (of [48]) (4/31, 12.9%), three of which were from North Inlet and one from Charleston Harbor. Two individuals of *A. ornata* were infected with *C. parvus* (2/55, 3.6%), both from North Inlet. These infections were observed in early to late fall (September/October) and none in winter. None of the 89 specimens of *S. hartmanae* were infected (all collected from Charleston Harbor). Six individuals of *P. palmata* were infected with developmental stages of an unknown blood fluke (6/29, 20.7%). All infected specimens of *P. palmata* were found in Charleston Harbor in late January 2026.

### Sequencing results for the parasites and phylogenetic analysis of the new blood fluke

Blood fluke sequences obtained from specimens collected from annelid hosts of the same species were identical to each other. LSU rRNA gene sequences (*n* = 4) of blood flukes found infecting *E. sanguineus* were 96% similar to those of *Neospirorchis* sp. CT-2017 (GenBank accession number LT882716, 97% coverage) and 95% similar to *N. chapmanae* (OQ702491, 100% coverage). COI (*n* = 3) and ITS2 (*n* = 2) sequences were 99% similar to those of *Neospirorchis* sp. Neogen14 (MH539821 [44% coverage] and MH539822 [100% coverage], respectively). LSU rRNA gene sequences of blood flukes from *A. ornata* (*n* = 3) were 99.8% similar to *C. parvus* (KY817377).

**Figure 1:**
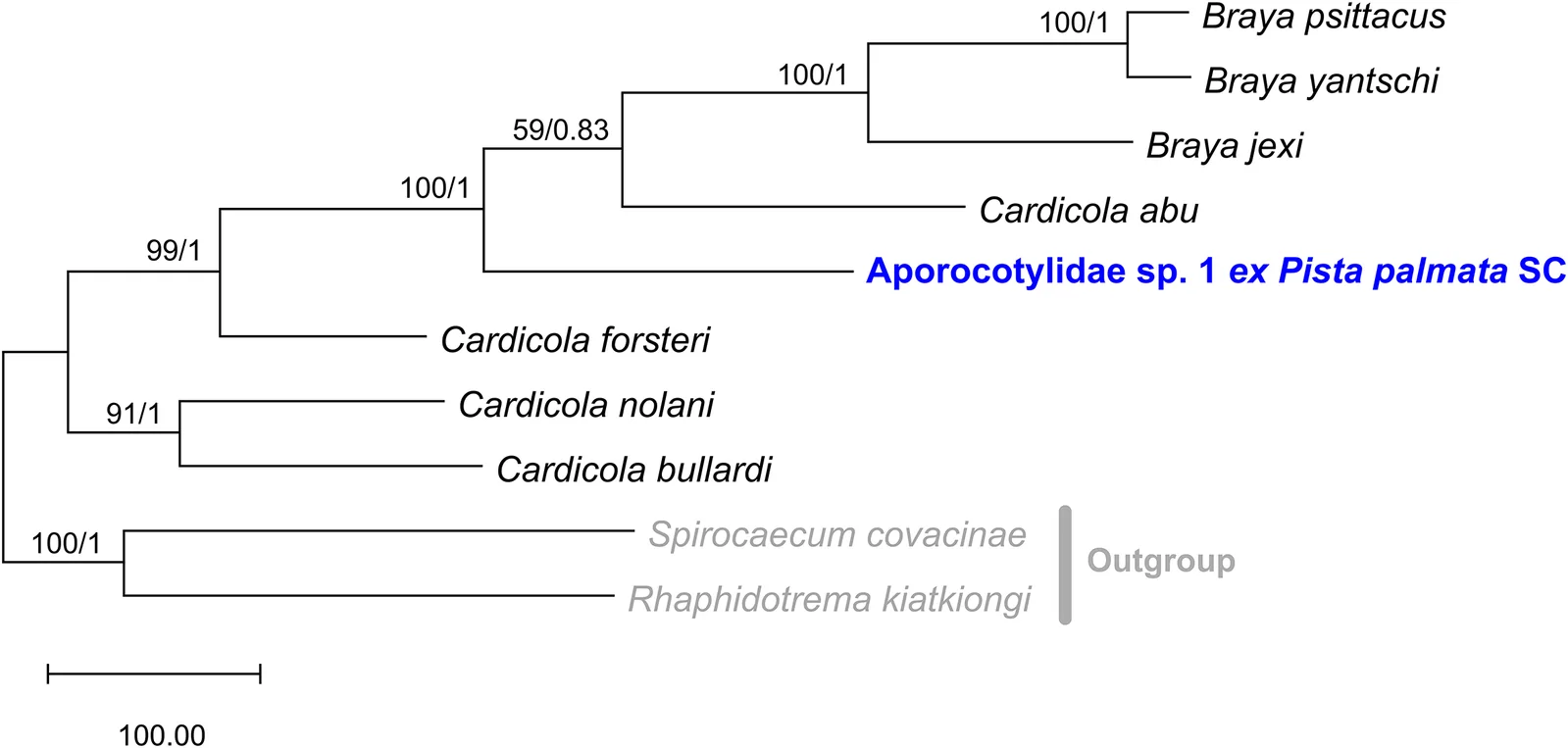
Maximum parsimony phylogenetic tree based on a 3,453-base pair concatenated alignment of large subunit ribosomal RNA (rRNA), second internal transcribed spacer region rRNA, and mitochondrial cytochrome *c* oxidase I gene sequences. Numbers at nodes indicate bootstrap support (1,000 replicates) followed by posterior probabilities from Bayesian analyses. See Table 3 for GenBank accession numbers used.

Five out of the six infected specimens of unknown blood flukes were successfully sequenced. Sequences from all three markers of the blood flukes collected from *P. palmata* (LSU [*n* = 2], ITS2 [*n* = 4], and COI [*n* = 2]; Table 2) were > 95% similar to those in GenBank, being most similar to sequences of *Braya* Nolan & Cribb, 2006 [38] and *Cardicola* (Tables 3 and 4). Based on *p*-distance calculations, the LSU rRNA gene sequence was most similar to *Braya jexi* Nolan & Cribb, 2006 [38], the ITS2 sequence was most similar to *C. abu* Yong, Cutmore & Cribb, 2018 [61], and the COI sequence was most similar to *B. psittacus* Nolan & Cribb, 2006 [38] (Table 4)*.* Maximum parsimony and Bayesian analyses yielded trees with similar topologies. Sequences from the new blood fluke specimens appeared in a well-supported clade (bootstrap support = 99, posterior probability = 1) with sequences from both *Braya* and *Cardicola* (Fig. 1). Given the overlap in *p*-distances and phylogenetic position of the new blood fluke sequences among two different genera, we hereafter refer to this blood fluke as Aporocotylidae sp. 1 *ex Pista palmata* SC.

**Table 4:** Number of base differences per site between the large subunit ribosomal RNA (LSU rRNA), second internal transcribed spacer region (ITS2) rRNA, and mitochondrial cytochrome *c* oxidase I (COI) gene sequences of the new blood fluke and those of described species. The number of base pairs (bp) analyzed for each gene region is in parentheses.

	*p*-distances		
Species	LSU (1,188 bp)	ITS2 (464 bp)	COI (393 bp)
*Braya jexi*	0.047	0.130	0.276
*Braya psittacus*	0.057	0.139	0.250
*Braya yantschi*	0.054	0.145	0.263
*Cardicola abu*	0.053	0.010	0.273
*Cardicola bullardi*	0.076	0.195	0.296
*Cardicola forsteri*	0.052	0.129	0.265
*Cardicola orientalis* ^*^	0.076	0.186	0.300
*Cardicola nolani*	0.064	0.171	0.263
*Rhaphidotrema kiatkiongi* ^†^	0.109	0.208	0.283
*Spirocaecum covacinae* ^†^	0.101	0.237	0.253

^*^Included only in alignments used to calculate *p*-distances. ^†^Used as outgroup sequences for phylogenetic analyses.

### Sequencing results for the new annelid host

COI sequences from the six specimens of new terebellid host were identical to one another (517–634 bp) and were 99.5% similar to sequences of *P. palmata* (OQ322725) with 97–100% query coverage.

### Morphological study of sporocysts and cercariae of Aporocotylidae sp. 1 ex *Pista palmata* SC

**Figure 2:**
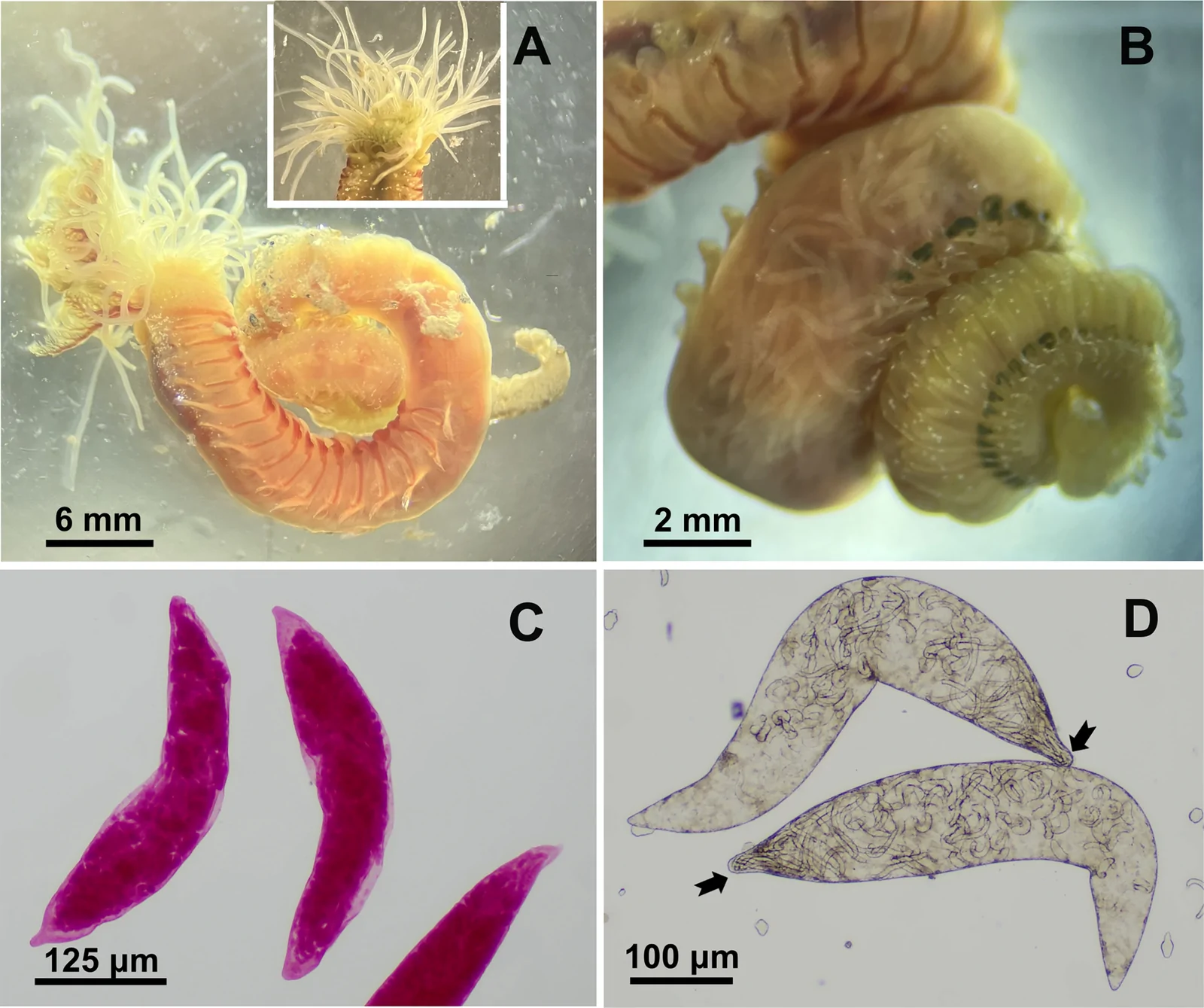
Aporocotylidae sp. 1 *ex Pista palmata* SC*.* A) Annelid host *P. palmata* with ventral view of prostomium (insert)*.* B) Infected individual *P. palmata* showing numerous sporocysts in hemocoel. C) Whole mount of sporocystogenous sporocysts (acetocarmine). D) Fresh wet mount of cercariogenous sporocysts. Arrows show birth pore.

**Figure 3:**
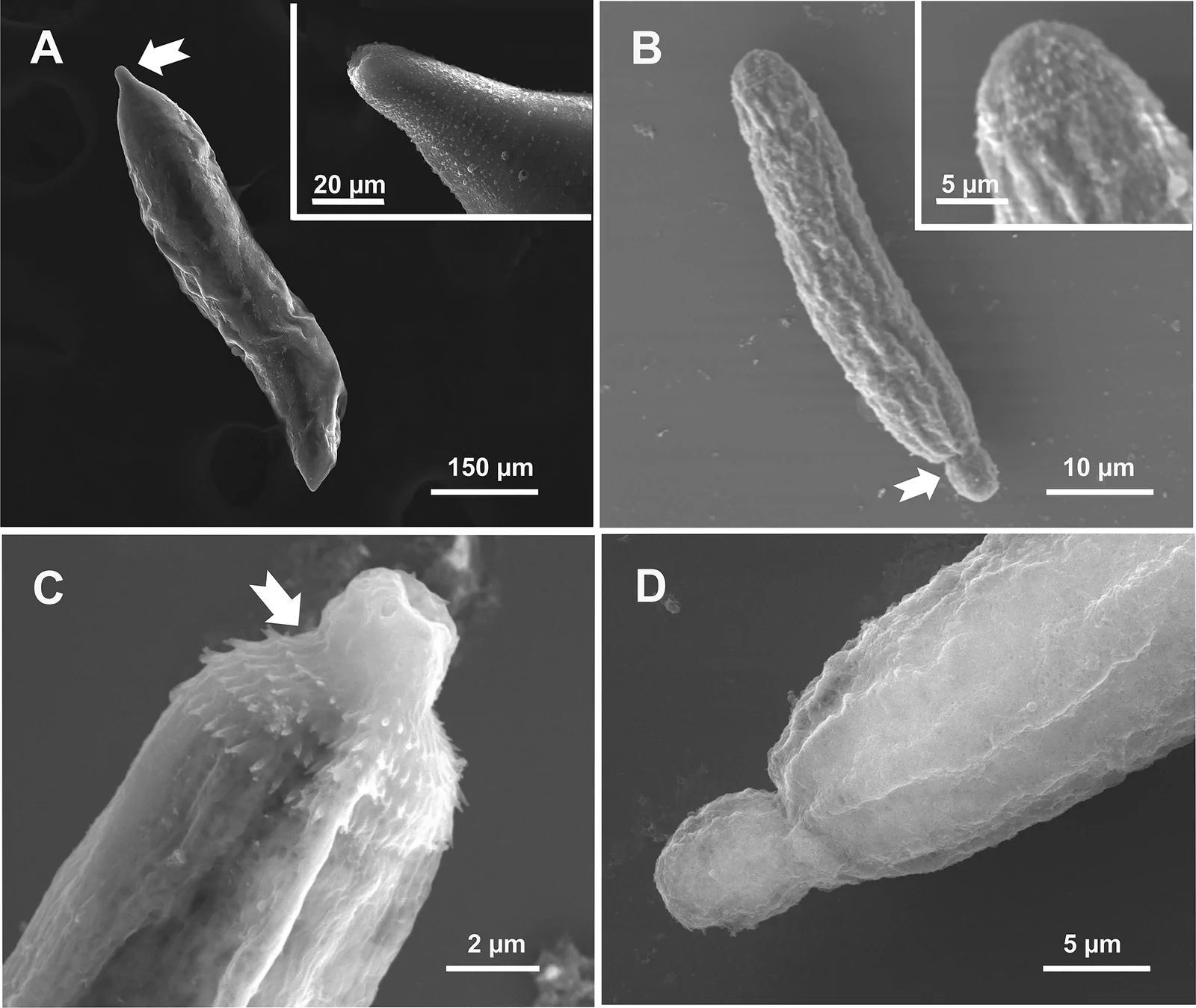
Scanning electron micrographs of sporocysts and cercariae of Aporocotylidae sp. 1 *ex Pista palmata* SC. A) Sporocyst with birth pore at extremity (arrow and insert). B) Microcercous cercaria with spheroid tail (arrow) and spinose anterior end (insert). C) Spinose anterior end showing aspinose extremity stretched in active cercaria. D) Spheroid tail end of cercaria.

**Figure 4:**
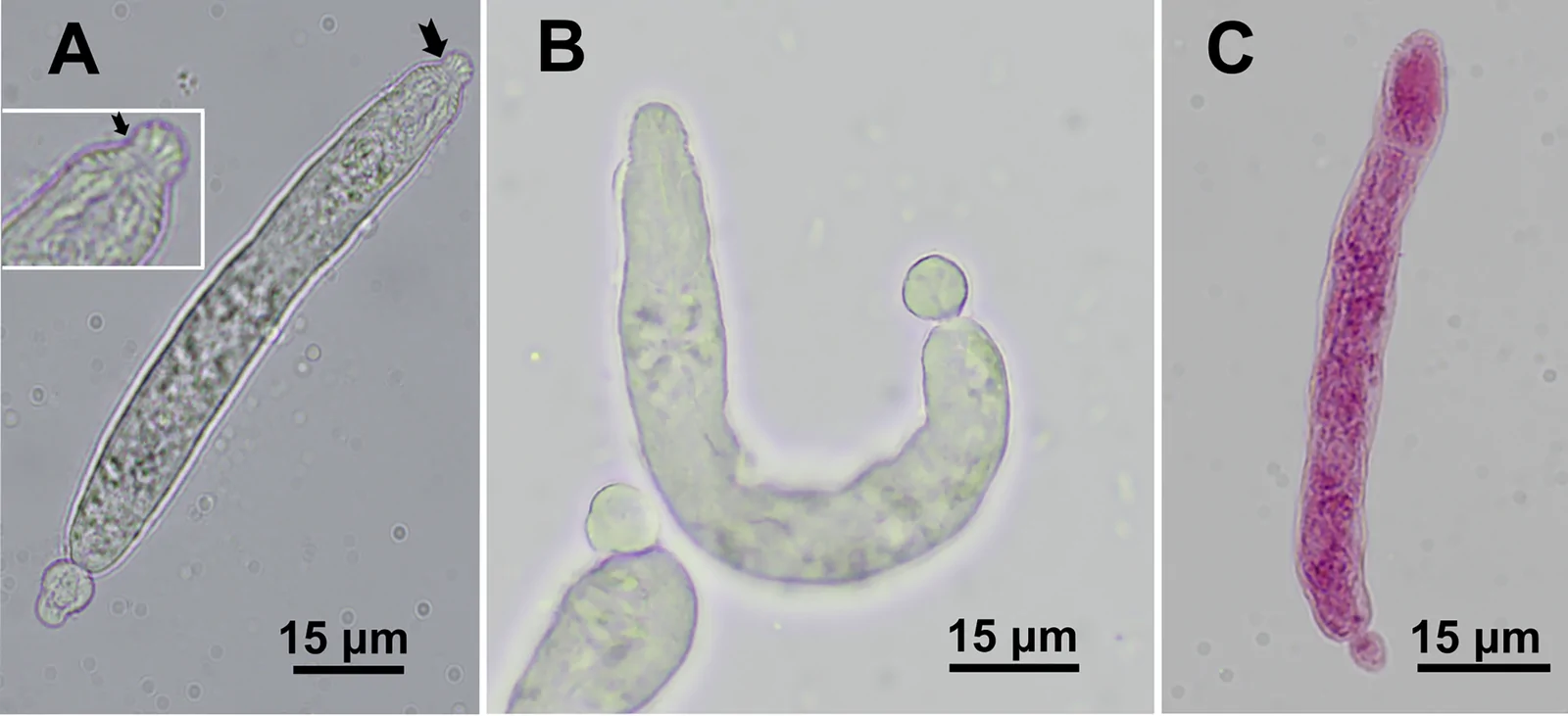
Microcercous cercariae of Aporocotylidae sp. 1 *ex Pista palmata* SC. A) Wet mount of cercaria actively moving showing a pyriform tail and rows of spines on anterior end (insert) with constriction (arrow) posterior to stretched aspinose extremity. B) Wet mount of heat-killed cercaria showing spheroid tail. C) Whole mount of cercaria (acetocarmine).

In all infected specimens of *P. palmata* (Fig. 2A), the very numerous sporocysts were floating free in the hemocoel (Fig. 2B) and were active when released into a Petri dish containing fresh seawater. Sporocysts were smooth, fusiform with a conspicuous terminal birth pore (Figs. 2C, 3A insert), and within each infected annelid, sporocysts at various stages of development were observed, with some being sporocystogenous (Fig. 2C) and others cercariogenous (Fig. 2D). Fixed immature sporocysts measured ~325 × 55 μm (*n* = 17) (length range: 150–425 μm) and mature sporocysts measured ~590 × 94 μm (*n* = 25) (length range: 500–775 μm). Cercariae in mature sporocysts were too numerous to be counted accurately (Fig. 2D). Emergence of active cercariae was observed from sporocysts upon cover-slipping*.* Cercariae (Figs. 3, 4) stretched and contracted their bodies but did not swim *per se* and remained at the top of the Petri dish, displaying a limited range of motion of their short tail, which changed from a pyriform (Fig. 4A) to a spheroidal shape (Figs. 3B, 3D, 4B) upon release from sporocysts. Cercariae showed on their anterior end 4–5 (*n* = 4) rows of minute spines (Figs. 3B, 3C, 4A insert), with the anteriormost row marking the presence of an aspinose extremity that appears to stretch out in active specimens (Figs. 3B insert, 3C, 4A). Cercariae were non-acetabulate, apharyngeate, non-ocellate, and microcercous with an elongated and tubular body that measured 80 × 13 μm (*n* = 6) and a spheroidal tail that measured ~5 × 5 μm (*n* = 6) in fixed specimens (Figs. 3B, 3D, 4C).

## Discussion

Information about the natural history of aporocotylids is scarce relative to the number of species described, with most known from economically important or aquacultured fishes [41]. One common characteristic of the few aporocotylid life cycles that have been elucidated thus far is that all use terebellid annelids*,* commonly known as “spaghetti worms”, which are tube-forming sedentary polychaetes and surface-deposit feeders, occupying various marine habitats, including cold seeps, intertidal waters, and hydrothermal vents [21]. *Pista* is a speciose genus that requires taxonomic revision and occurs worldwide [22]. The distribution of *P. palmata* however, appears to be restricted to the Gulf of Mexico, the northwestern Atlantic Ocean along the United States coast, and the Caribbean Sea off Puerto Rico (https://copepedia.org/?id=T4064070). Specimens of *P. palmata* were examined in 1945 in the northeastern USA (Woods Hole area), but no infection by blood flukes was observed [42]. We found our specimens of *P. palmata* in muddy tubes encrusted underneath hard surfaces of rocks in the intertidal zone of Charleston Harbor. Sporocysts of the newly found blood flukes were unremarkable. However, the cercariae, being microcercous, were different from others that we have observed thus far from annelids on the SC coast [16, 47], and most closely resemble those of *C. orientalis* (see [45]), an aporocotylid that infects various tuna such as the Pacific bluefin tuna *Thunnus orientalis* Temminck & Schlegel and the Atlantic bluefin tuna *T. thynnus* (L.) (see [17, 40, 49]). However, molecularly, the two species are not closely related, as the LSU rRNA gene sequence of *C. orientalis* was only 92.4% similar to the new blood fluke, and ITS2 and COI sequences only 81.4% and 70.0% similar, respectively*.* This cercarial morphotype has not been documented in the Atlantic Ocean, other than for *Cercaria solemyae* Martin, 1944 [32] that was collected from the Atlantic awning clam *Solemya velum* Say*,* near Woods Hole, Massachusetts, USA and which was suspected to be a larval blood fluke at the time [32]. However, except for being about the same length and microcercous, the two cercariae are otherwise morphologically very different; particularly, cercariae of *C. solemyae* have a spinose body but seemingly lack the few rows of anterior spines that cercariae of Aporocotylidae sp. 1 *ex Pista palmata* SC have. Furthermore, although there are no molecular data from *C. solemyae* to support that the two are not closely related, this is likely the case as one infects an annelid and the other a bivalve. To our knowledge the only fish-infecting blood flukes that are described from bivalves are chimaerohemecids (blood flukes of chondrichthyans) and elopicolids (blood flukes of elopiform and anguilliform osteichthyans) [11, 15, 19, 56], which are distinct from aporocotylids [57]. Our phylogenetic analysis indicated that the closest relatives of the newly found blood fluke are aporocotylids *Braya* spp. and *C. abu*, species within the problematic *Cardicola* clade [62]*.* We were therefore unable to assign the new blood fluke to a genus within this clade. Significantly, all three *Braya* spp. and *C. abu*, whose sequences appeared in a well-supported phylogenetic clade with those of Aporocotylidae sp. 1 *ex P. palmata* SC, are parasites of reef fishes in Australia. *Braya* spp. infect parrotfishes (*Scarus frenatus* Lacepède*, S. ghobban* Forsskål*,* and *Chlorurus microrhinos* Bleeker), and *C. abu* infects the damselfish *Abudefduf whitleyi* Allen & Robertson (see [38]). As aporocotylids are considered stenoxenous [41, 43, 63], the definitive host of our specimens, therefore, may also be a reef fish, possibly in the family Pomacentridae (damselfishes) or Labridae (wrasses and parrotfishes)*,* which are quite diverse and common in the Charleston Harbor area ([24], reef.org)*.* Given our understanding of the chronobiology of cercarial release, albeit in mollusks [52], and because specimens of *P. palmata* were infected with mature sporocysts in the winter at > 20% prevalence, we expect that the definitive host or hosts inhabit our coasts during that time, thus ensuring transmission.

In conclusion, we discovered a novel aporocotylid developing in a terebellid that had not previously been reported as an intermediate host of blood flukes. Although this finding expands our knowledge of the diversity of aporocotylids on the Atlantic coast of the USA, the species reported herein has yet to be found in its fish definitive host in order to be named. Further, gaps still remain in the identity of the intermediate hosts of most of the aporocotylid species reported from their fish definitive hosts, and these life cycles have yet to be elucidated. Considering that SC is part of the Grand Caribbean Region, which is home to numerous species of terebellids [31], it is likely that other terebellid species serve as intermediate hosts of yet-to-be-discovered blood flukes.
